# Trend and burden of ischemic stroke attributable to high body mass index in China from 1990 to 2021, with projection to 2044

**DOI:** 10.1371/journal.pone.0335616

**Published:** 2025-12-04

**Authors:** Ning Ding, Ruiwen Fan, Xiaohui Zhang, Jiacheng Zhang, Yutian Zhu, Xiyan Xin, Yang Ye

**Affiliations:** 1 Department of Traditional Chinese Medicine, Peking University Third Hospital, Beijing, China; 2 Department of Traditional Chinese Medicine, Peking University Third Hospital Qinhuangdao Hospital, Qinhuangdao, China; UCSF: University of California San Francisco, UNITED STATES OF AMERICA

## Abstract

**Background:**

Ischemic stroke (IS) poses a significant public health burden in China, with high body mass index (BMI) identified as a key risk factor. Despite this, the temporal trends and sex-specific burdens of IS attributable to high BMI remain insufficiently explored. This study examines the burden of IS linked to high BMI in China from 1990 to 2021 and projects future trends to 2044.

**Methods:**

Drawing on data from the Global Burden of Disease Study 2021, we first employed correlation analysis to examine the association between high BMI exposure and IS incidence in China over a 30-year period. Subsequently, we quantified the mortality and disability-adjusted life years (DALYs) attributable to high BMI-related IS. Our analysis included comparative assessments with global and high-income country trends, complemented by sex-stratified and age-specific evaluations. Finally, we developed forecasting models to project disease burden trajectories through 2044.

**Results:**

From 1990 to 2021, China’s age-standardized IS incidence initially rose before stabilizing, with males consistently exhibiting higher rates than females. High BMI exposure increased steadily and strongly correlated with IS incidence. Age-standardized mortality attributable to high BMI grew from 0.98 to 2.21 per 100,000, while DALYs more than doubled from 24.61 to 56.21 per 100,000, with males disproportionately affected. The elderly, particularly men, showed the steepest increases. Unlike global declines, China’s high BMI-attributable burdens rose. Projections suggest continued growth, reaching 4.05 (male) and 3.23 (female) mortality rates by 2044, with DALYs at 101.33 and 96.63 per 100,000 respectively.

**Conclusion:**

High BMI contributes to a growing and sex-disparate burden of IS in China, with accelerated growth in older adults. Unlike high-income countries, China has not yet achieved declines in high BMI-attributable IS mortality and DALYs. Urgent public health interventions targeting BMI reduction, particularly in males and older populations, are needed to mitigate future burdens.

## Introduction

Ischemic stroke (IS) poses a significant global public health challenge, characterized by high morbidity, mortality, and disability rates [1]. As a leading cause of death and disability-adjusted life years (DALYs) worldwide, IS affects millions of individuals, with substantial socioeconomic impacts [2]. The condition arises from cerebral blood flow interruption, leading to neuronal damage and functional impairment [3]. Its epidemiology is shaped by multiple risk factors, including non-modifiable factors like age and genetics, as well as modifiable factors such as hypertension, diabetes, and obesity [4].

In China, IS has become a critical public health issue, contributing significantly to the national disease burden [5]. The country’s large population, rapid aging, and shifting lifestyle patterns have driven changes in IS incidence and risk factor profiles. While previous studies have highlighted the growing burden of IS in China, the specific role of high body mass index (BMI) as a risk factor remains under-explored in terms of temporal trends, sex-specific differences, and age-distributed impacts [6–9]. High BMI is a well-recognized risk factor for IS, linked to mechanisms such as atherosclerosis, insulin resistance, and inflammation [10]. Globally, increasing obesity rates have been associated with rising IS burdens, but regional and sex-specific patterns vary [11]. In China, the prevalence of high BMI has increased notably in recent decades, paralleling economic development and dietary transitions [12]. However, limited research has quantified the burden of IS attributable to high BMI in the Chinese population, particularly through a sex- and age-stratified lens and in comparison with global and high-income country trends.

This study aims to address these knowledge gaps by analyzing the burden of IS linked to high BMI in China from 1990 to 2021, with projections to 2044. Using data from the Global Burden of Disease (GBD) Study 2021, we examine trends in IS incidence, mortality, and DALYs attributable to high BMI, with a focus on sex and age differences. By integrating comparative analyses with global and high-income country data and developing forecasting models, this research seeks to provide insights into the evolving role of high BMI in China’s IS epidemic and inform targeted public health interventions.

## Methods

### Data sources

We obtained data on age-specific and age-standardized mortality rates and DALY rates for IS attributable to high BMI in China and globally from 1990 to 2019 from the GBD Study 2021 database (https://vizhub.healthdata.org/gbd-results/) [13]. GBD 2021 is a publicly accessible database, and descriptions of the methods used to evaluate risk factor data have been previously published [14]. This study was conducted in strict accordance with the Guidelines for Accurate and Transparent Health Estimation Reporting for Population Health Research [15]. As the GBD dataset does not contain any personally identifiable information, ethical review and informed consent were not required for this study [16]. The study population was stratified into 15 age cohorts spanning from 25–29 to 90–94 years, with an additional category for individuals aged ≥95 years. Age-standardized incidence rates were computed by applying the GBD 2021 reference population structure.

### Case definition

Stroke, as defined by the World Health Organization diagnostic criteria, manifests as an acute onset of neurological deficits indicative of cerebral dysfunction, persisting beyond 24 hours or leading to fatal outcomes [17]. Under the GBD 2021 classification framework, IS is conceptualized as a cerebrovascular event characterized by cerebral hypoperfusion and resultant infarction [18]. This category includes thromboembolic and atherosclerotic etiologies but strictly excludes hemorrhagic stroke. Case identification utilized the International Classification of Diseases, Tenth Revision (ICD-10) codes G45–G46.8 (transient cerebral ischemic attacks and related syndromes), I63–I63.9 (cerebral infarction), I65–I66.9 (occlusion and stenosis of precerebral/cerebral arteries), I67.2–I67.8 (specific cerebrovascular disorders), and I69.3–I69.4 (sequelae of cerebral infarction) [8].

### High BMI

High BMI is operationally defined as a BMI ≥ 23 kg/m² in adults aged ≥20 years [1]. The GBD 2021 incorporated high BMI estimates derived from systematically collated data sources, including population-based surveys, epidemiological studies, and clinical records. Standardized exposure value (SEV) for risk factors is calculated by normalizing raw exposure data via age-standardization to enable cross-regional and temporal comparisons, with specifics varying by factor in GBD’s technical reports. Methodological details regarding source selection criteria, input data curation, and quality control procedures are comprehensively documented in prior GBD publications [19].

In GBD, the estimation of stroke mortality attributable to high BMI involves a systematic approach. First, associations between high BMI and stroke subtypes are identified. Then, relative risks are evaluated as functional exposures based on comprehensive systematic reviews. Next, high BMI exposure is estimated according to age, sex, region, and year, followed by the determination of the theoretical minimum risk exposure level. Subsequently, attributable disease burden and population attributable fractions are calculated. Finally, to account for the complex interplay of risk factors, the number of attributable deaths from the combined effects of high BMI, high fasting plasma glucose, high low-density lipoprotein cholesterol, and high systolic blood pressure are estimated, taking into consideration the mediating effects of these risk factors [20].

### Key metrics

The key metrics involved in this study include incidence, mortality, DALYs, and high BMI exposure rate. The age-standardized mortality rate represents the number of IS deaths attributable to high BMI per 100,000 population after age adjustment. The DALYs rate is a composite metric integrating years of life lost due to premature mortality and years lived with disability due to disability, also age-standardized to reflect the overall disease burden [21]. These metrics were analyzed for their changes from 1990 to 2021.

### Statistical analysis

Data on age-specific and age-standardized mortality rates, DALYs, and high BMI exposure were extracted from the GBD 2021 database. Age standardization was performed using the GBD 2021 reference population structure to facilitate temporal and regional comparisons. The association between high BMI exposure and IS incidence in China (1990–2021) was assessed using linear regression analysis, stratified by sex (male/female) and overall population, with statistical significance defined as *P* < 0.05. Subgroup analyses were conducted by sex and 15 age groups (25–29 years to ≥95 years) to evaluate age-specific burden trends. For future projections, we employed an autoregressive integrated moving average (ARIMA) model to forecast mortality and DALY rates up to 2044 based on historical data from 1990 to 2021 [22]. The optimal ARIMA parameters were selected by minimizing the Akaike Information Criterion (AIC) and Bayesian Information Criterion (BIC), with model diagnostics including the Ljung–Box test for residual autocorrelation. To account for model variability, uncertainty intervals (80% and 95%) were estimated for predicted values.

## Results

### Trends and correlation between IS incidence and High BMI exposure in China

**Fig 1:**
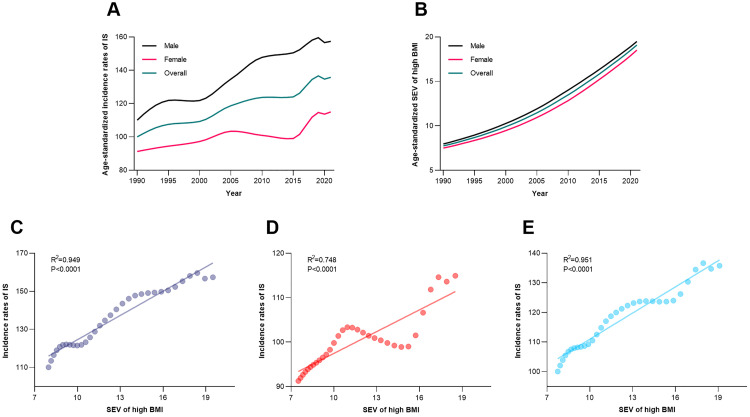
Trends and correlation of age-standardized incidence of IS and high BMI exposure in China, 1990–2021. (A) Age-standardized incidence of IS (per 100,000) by sex and overall. (B) Age-standardized exposure rate of high BMI (%) by sex and overall. (C-E) Scatter plots illustrating the correlation between high BMI exposure and IS incidence in males, females, and the total population. BMI, body mass index; IS, ischemic stroke; SEV, standardized exposure value.

From 1990 to 2021, China’s age-standardized incidence of IS initially increased and then stabilized, with the combined rate rising from 100.05 per 100,000 in 1990 to 135.79 per 100,000 in 2021 (Fig 1). Males consistently had higher rates than females, peaking at 159.59 per 100,000 in 2019 for males and 114.62 per 100,000 in 2019 for females. Concurrently, the age-standardized exposure rate of high BMI showed a continuous upward trend, increasing from 7.75% in 1990 to 19.09% in 2021, with males always having higher exposure than females. Correlation analysis revealed a strong positive association between high BMI exposure and IS incidence in males (R² = 0.949, *P* < 0.0001), females (R² = 0.748, *P* < 0.0001), and the overall population (R² = 0.951, *P* < 0.0001), suggesting a strong association between high BMI and IS incidence (Fig 1).

### Age-standardized death and DALYs rates for IS attributable to high BMI in China

**Fig 2:**
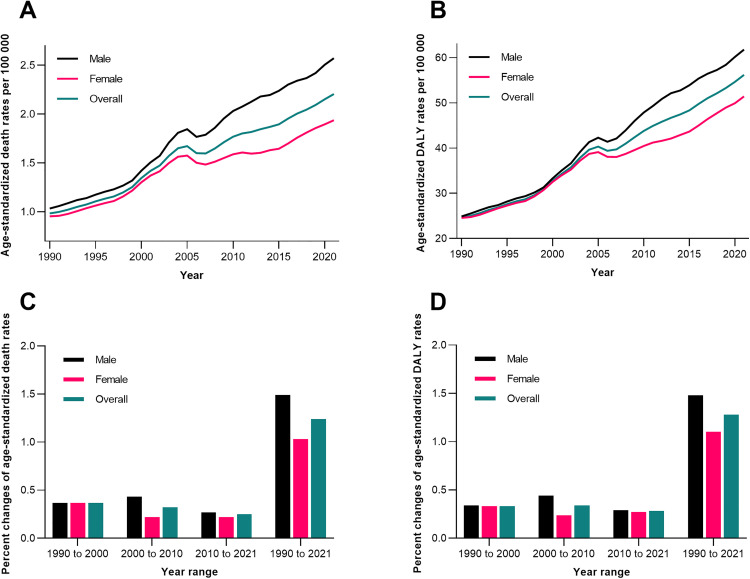
Age-standardized rates of death and DALYs for IS attributable to high BMI in China, 1990–2021. (A) Death rates (per 100,000) by sex and overall. (B) DALYs rates (per 100,000) by sex and overall. (C) Average change in death rates across periods (1990–2000, 2000–2010, 2010–2021). (D) Average change in DALYs rates across periods. BMI, body mass index; DALYs, disability-adjusted life years; IS, ischemic stroke.

From 1990 to 2021, China’s age-standardized death rates for IS attributable to high BMI increased consistently, rising from 0.98 per 100,000 (95% CI: 0.12–1.99) in 1990 to 2.21 per 100,000 (95% CI: 0.30–4.58) in 2021, with males always higher than females (Fig 2; Table 1). DALYs rates showed a similar upward trend, growing from 24.61 per 100,000 (95% CI: 3.09–50.09) to 56.21 per 100,000 (95% CI: 7.84–113.00). Subgroup analysis revealed distinct period trends: death rates grew fastest during 1990–2000 and 2000–2010 (0.43 per 100,000, 95% CI: 0.21–0.73), while post-2010 growth slowed (0.25 per 100,000, 95% CI: 0.01–0.49). DALYs rates exhibited sustained growth across all periods, with the highest increase in 1990–2021 (1.28 per 100,000, 95% CI: 0.82–1.94). Male-specific rates consistently exceeded female rates across all periods, reflecting a persistent sex disparity in the burden of high BMI-related IS (Fig 2).

**Table 1:** Age-standardized death and DALYs rates for IS attributable to high BMI in China, 1990-2021.

	Age-standardized death rate, per 100,000 (95% UI)			Age-standardized DALYs rate, per 100,000 (95% UI)		
	1990	2021	Change (%)	1990	2021	Change (%)
Male	1.03 (0.13 to 2.12)	2.57 (0.35 to 5.39)	148.7 (72.9 to 257.9)	24.89 (3.01 to 49.38)	61.78 (8.61 to 126.27)	148.3 (77.7 to 250.2)
Female	0.95 (0.12 to 1.98)	1.94 (0.26 to 3.93)	103.1 (44.8 to 186.7)	24.56 (3.19 to 49.01)	51.45 (7.25 to 102.18)	109.5 (58.6 to 182.5)
Both	0.98 (0.12 to 1.99)	2.21 (0.3 to 4.58)	124.2 (71 to 197.2)	24.61 (3.09 to 50.09)	56.21 (7.84 to 113)	128.4 (82.3 to 193.9)

BMI, body mass index; DALYs, disability-adjusted life years; IS, ischemic stroke; UI, uncertainty interval.

### Age-specific death and DALYs rates for IS attributable to high BMI in China

**Fig 3:**
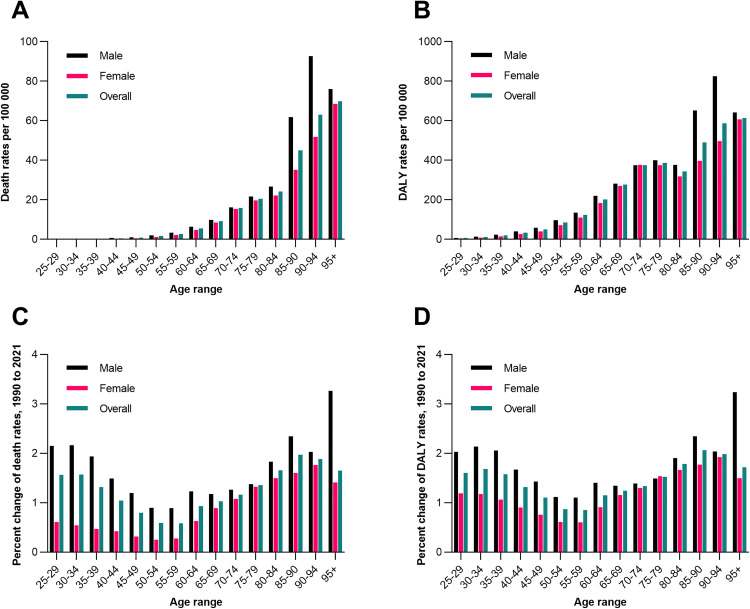
Age-specific rates of death and DALYs for IS attributable to high BMI in China, 1990-2021. (A) Death rates (per 100,000) by sex and age in 2021. (B) DALYs rates (per 100,000) by sex and age in 2021. (C) Average change in death rates by age group (1990–2021). (D) Average change in DALYs rates by age group (1990–2021). BMI, body mass index; DALYs, disability-adjusted life years; IS, ischemic stroke.

In 2021, age-specific death rates for IS attributable to high BMI in China exhibited a clear upward trend with age across both sexes, ranging from 0.05 per 100,000 (95% CI: 0.01–0.11) in males aged 25–29 to 92.6 per 100,000 (95% CI: 11.74–204.43) in males aged 90–94, and from 0.02 per 100,000 (95% CI: 0.00–0.04) in females aged 25–29 to 68.46 per 100,000 (95% CI: 8.40–163.10) in females aged >95 (Fig 3). DALYs rates showed a similar pattern, with the lowest rate in males aged 25–29 (6.7 per 100,000, 95% CI: 1.00–13.00) and the highest in males aged 90–94 (825.64 per 100,000, 95% CI: 104.93–1821.76). Females consistently had lower rates than males across all age groups, though the sex gap narrowed in older age brackets. Mortality rates exhibited a J-shaped curve with age, peaking in individuals over 95 years (3.26 per 100,000 in males, 1.41 per 100,000 in females). Younger adults (25–39 years) experienced the highest mortality increases (1.93–2.16 per 100,000 in males; 0.47–0.61 per 100,000 in females), while older age groups (≥80 years) showed accelerated growth, particularly among males. DALYs mirrored this trend, with the steepest rise in the 85–90 age group (489.55 per 100,000 overall), driven predominantly by male burdens (2.35 vs. 1.77 per 100,000 in females). Notably, the male-to-female disparity narrowed after age 80, though males consistently bore higher burdens across all ages (Fig 3).

### Trends in IS incidence, high BMI exposure, and attributable mortality/DALYs in global, China, and high-income countries

**Fig 4:**
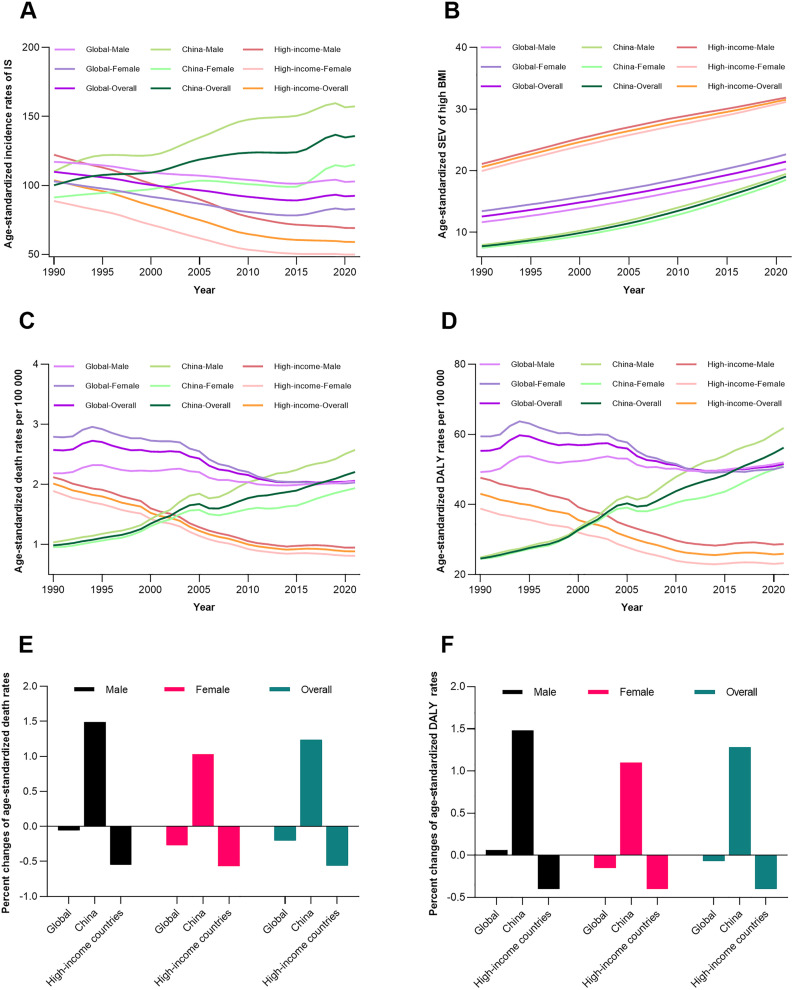
Trends in IS incidence, high BMI exposure, and high BMI-attributable mortality/DALYs in global, China, and high-income country populations, 1990–2021. (A) Age-standardized incidence of IS (per 100,000) by sex and region. (B) Age-standardized high BMI exposure rates by sex and region. (C) Age-standardized death rates (per 100,000) for IS attributable to high BMI by sex and region. (D) Age-standardized DALYs rates (per 100,000) for IS attributable to high BMI by sex and region. (E-F) Average change in death rates and DALYs rates by region and sex over the entire period. BMI, body mass index; DALYs, disability-adjusted life years; IS, ischemic stroke.

From 1990 to 2021, global age-standardized IS incidence declined slightly in males (from 117.02 to 102.77 per 100,000) and females (from 102.87 to 82.85 per 100,000), with a similar trend in high-income countries (males: 122.14 to 68.97; females: 88.65 to 49.72; Fig 4). In contrast, China experienced an initial increase in incidence until the mid-2010s, stabilizing thereafter (males: 110.06 to 157.38; females: 91.25 to 114.93). High BMI exposure rates rose consistently worldwide, with China showing the steepest growth (from 7.75% to 19.09%), surpassing high-income countries (20.58% to 31.88% in males; 19.96% to 31.17% in females). Mortality and DALYs attributable to high BMI followed similar patterns: China’s age-standardized death rates increased from 0.98 to 2.21 per 100,000 (males: 1.03 to 2.57; females: 0.95 to 1.94), while high-income countries saw declines (from 2.01 to 0.88 per 100,000 in males; 1.89 to 0.81 in females). Global trends reflected mixed patterns, with slight mortality decreases offset by DALYs stability. Sex disparities persisted, with males consistently bearing higher burdens across all regions (Fig 4).

### Age-specific death and DALYs rates for IS attributable to high BMI in global, China, and high-income countries

**Fig 5:**
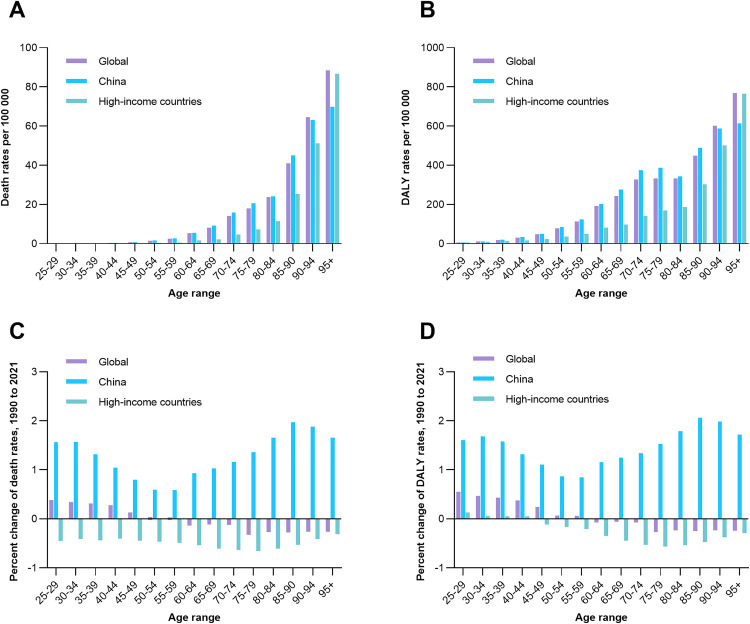
Age-specific death and DALYs rates for IS attributable to high BMI in global, China, and high-income country populations. (A) Death rates (per 100,000) by age and region in 2021. (B) DALYs rates (per 100,000) by age and region in 2021. (C) Average percentage change in death rates by age group (1990–2021). (D) Average percentage change in DALYs rates by age group (1990–2021). BMI, body mass index; DALYs, disability-adjusted life years; IS, ischemic stroke.

In 2021, age-specific death rates for IS attributable to high BMI increased with age across all regions, with the highest rates observed in the oldest age groups (≥85 years; Fig 5). China’s death rate for individuals aged >95 years was 69.87 per 100,000 (95% CI: 8.70–165.58), lower than the global rate of 88.47 per 100,000 (95% CI: 11.22–199.57) and high-income countries’ 86.67 per 100,000 (95% CI: 10.91–197.01). DALYs rates followed a similar pattern, with China’s 85–90 age group showing the highest burden at 489.55 per 100,000 (95% CI: 62.96–1,078.00), surpassing high-income countries (302.82 per 100,000, 95% CI: 38.65–666.06) and the global average (447.59 per 100,000, 95% CI: 59.57–993.00). While high-income countries had higher DALYs rates in younger age groups (e.g., 25–29 years: 5.84 vs. China’s 6.09), China’s older age groups (≥80 years) exhibited faster relative growth. From 1990 to 2021, global death rates in younger age groups (25–49 years) increased slightly, while older age groups showed declines. China experienced significant growth in death rates across all age groups, particularly in those aged 25–39 years, whereas high-income countries showed consistent declines in older age groups. DALYs rates in China grew rapidly in older age groups, while high-income countries showed stability or declines, especially in those aged ≥60 years (Fig 5).

### Predicted trends in death and DALYs rates for IS attributable to high BMI in Chinese males and females, 2022–2044

**Fig 6:**
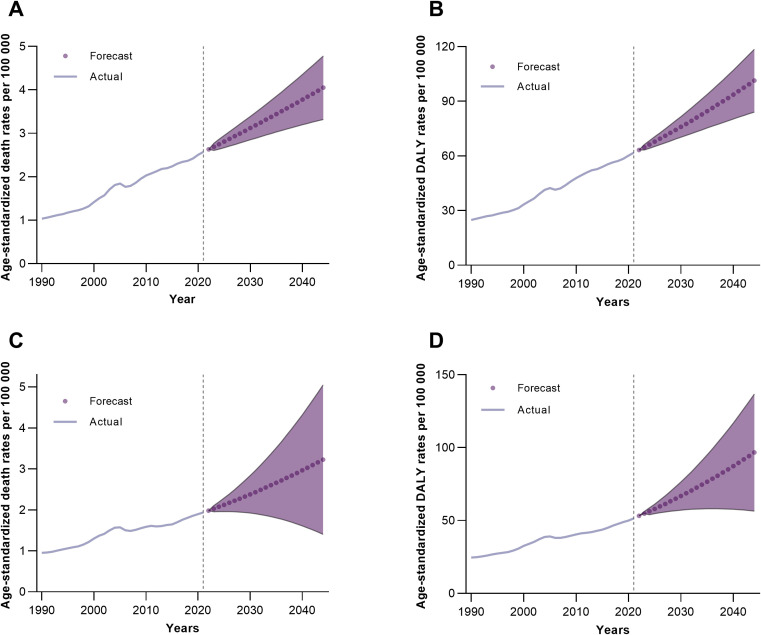
Predicted trends in death and DALYs rates for IS attributable to high BMI in Chinese populations. (A) Male death rates. (B) Male DALYs rates. (C) Female death rates. (D) Female DALYs rates. BMI, body mass index; DALYs, disability-adjusted life years; IS, ischemic stroke.

The forecasts show that death rates for IS attributable to high BMI in Chinese males are projected to increase from 2.63 per 100,000 in 2022 to 4.05 per 100,000 by 2044, with an 80% prediction interval ranging from 3.32 to 5.05 and a 95% interval from 2.93 to 6.02 (Fig 6). For females, the rates are expected to rise from 1.98 per 100,000 in 2022 to 3.23 per 100,000 by 2044, with 80% and 95% intervals spanning 1.40–5.05 and 0.44–6.02, respectively. DALYs follow a similar upward trajectory: males are predicted to increase from 63.23 per 100,000 in 2022 to 101.33 per 100,000 in 2044 (80% CI: 84.10–118.57; 95% CI: 74.97–127.70), while females are projected to rise from 53.14 per 100,000 to 96.63 per 100,000 (80% CI: 56.44–136.82; 95% CI: 35.17–158.10). Both sexes exhibit consistent growth across the forecast period, with males consistently having higher rates than females (Fig 6).

## Discussion

This study systematically evaluated the disease burden of IS caused by high BMI in China from 1990 to 2021 and predicted its development trend up to 2044. The analysis shows that the number of deaths and DALYs related to high BMI have shown a continuous upward trend with gender differences, which stands in stark contrast to the continuously declining trend in high-income countries. During the study period, the age-standardized mortality rate attributed to high BMI more than doubled, and the burden on males was consistently higher than that on females. Notably, the upward trend in disease burden was more pronounced among the elderly population, especially in males aged 80 years and above. Model predictions indicate that the mortality rate of high BMI-related IS in China may continue to rise over the next 20 years.

This study adopts a BMI cutoff of ≥23 kg/m² for defining high BMI, primarily based on the unique obesity-related health risk profile of Asian populations. This differs from the WHO’s ≥25 kg/m² threshold, which is derived from data on European populations [23]. Epidemiological studies show that when Asians have a BMI of 23–24.9 kg/m², their visceral fat accumulation rate, insulin resistance incidence, and metabolic syndrome risk are equivalent to those of Europeans with a BMI of 25–26.9 kg/m² [24]. This discrepancy stems from Asians’ higher propensity for “central obesity”—even without reaching the overall weight threshold, their abdominal fat proportion may still increase significantly [25].

In China, the increase in the incidence of IS shows a significantly synchronous trend with the continuous rise in high BMI exposure levels, with the correlation coefficients for males and the total population both exceeding 0.9. This strong correlation is highly consistent with the pathophysiological theory that obesity accelerates atherosclerosis and thrombosis through mechanisms such as chronic inflammation, endothelial dysfunction, and insulin resistance [26–28]. Gender differences may stem from biological variations in fat distribution and hormone levels: visceral fat, which males are more prone to accumulate, releases pro-inflammatory cytokines that directly damage blood vessels [29,30], while estrogen in premenopausal females exerts a protective effect by regulating lipid metabolism and endothelial function [31]. Notably, the narrowing of gender differences in the elderly population suggests that postmenopausal hormonal changes and cumulative metabolic damage may weaken this protective advantage over time [32]. The stabilization of IS incidence after 2010 may be related to China’s 2009 healthcare reform, which expanded the coverage of medical insurance funds to a broader population, better control of common chronic diseases such as hypertension and diabetes has helped reduce the risk of IS [33].

The doubling of the age-standardized mortality rate and DALY rate for high BMI-related IS may be attributed to the combined effects of factors such as changes in China’s population structure, socioeconomic development, and shifts in lifestyle over the past three decades. Amid China’s rapid urbanization, high-energy dietary patterns have replaced traditional dietary structures, while physical activity levels have continued to decline. Over the past 40 years, the prevalence of overweight/obesity has surged [34]. This behavioral transition has had a particularly notable impact on the male population—males have a higher incidence of unhealthy behaviors such as smoking and alcohol consumption, which interact synergistically with obesity to further amplify the risk of IS [35]. Notably, the increase in DALYs has outpaced that of the mortality rate, suggesting that obesity-related metabolic disorders may exacerbate the burden of long-term disability caused by stroke recurrence and sequelae [36].

The J-shaped mortality curve observed in specific age groups indicates that the mortality rate of high BMI-related IS is relatively higher in both young and elderly populations. The aging process exacerbates obesity-related vascular sclerosis and cerebral hypoperfusion, making the elderly population susceptible to ischemic injury even with a slight increase in BMI [37]. The sharp rise in mortality rate observed in the young and middle-aged group (25–39 years old) suggests a trend toward younger onset of stroke in China, which may be associated with the epidemiological data showing that the overweight rate and obesity rate among adolescents are 34.8% and 14.1%, respectively [38]. The narrowing of gender differences in the elderly age group may reflect the attenuation of the protective effect of estrogen in postmenopausal women [39].

A contrast exists between the trends in BMI-related IS burden in China and high-income countries—with the burden continuing to rise in the former and declining steadily in the latter—and this is associated with multiple factors. High-income countries have established obesity prevention and control networks through intervention measures such as sugar tax policies and mandatory calorie labeling, while China’s anti-obesity policies still need to be strengthened [40,41]. For instance, although Healthy China 2030 sets obesity control targets, it lacks provisions for food marketing or other specific measures [42]. At the clinical treatment level, high-income countries have significantly reduced stroke severity through innovations in neuroimaging technology and optimization of thrombolytic regimens, whereas the thrombolysis rate in some regions of China remains below 8% [43]. China still lags behind high-income countries in the prevention and control of high BMI-related IS, and continued efforts are needed across various sectors.

The abnormally high DALY rate among the elderly population in China reflects a potential gap between China’s post-stroke care system and that of high-income countries. High-income countries have effectively shortened disability cycles through comprehensive stroke unit management models and early discharge support programs, whereas China’s healthcare system still has a structural bias toward prioritizing acute-phase treatment over chronic-phase management [44]. Notably, although the rapid rise in the DALY rate among the young population aged 25–39 aligns with the global trend, the magnitude of increase is more pronounced—this may stem from the unique dual metabolic burden of coexisting malnutrition and obesity among low-income groups in the Asia-Pacific region [45]. Under the combined influence of Westernized dietary patterns, this metabolic vulnerability may further increase the risk of IS [46].

ARIMA model predictions indicate that the male stroke mortality rate will reach 4.05 per 100,000 by 2044, highlighting the still severe public health situation facing China. This prediction is based on the assumption of a linear trend in obesity rate growth and IS incidence. However, actual outcomes may deviate from expectations due to factors such as policy changes or other emerging variables. If China can achieve the goal of curbing obesity growth, the mortality rate may stabilize earlier [47]. The expanded prediction interval for females reflects greater variability in their lifestyle transitions—they are simultaneously burdened by the dual pressures of workplace competition and traditional family care responsibilities. The adoption of more complex modeling frameworks in the future—such as Bayesian models or machine learning methods—may enhance the accuracy of prediction results by integrating multiple covariates and capturing nonlinear dynamic relationships.

Several limitations should be acknowledged in this study. First, as this study is based on observational data and correlation analysis, it can only demonstrate associations between high BMI and ischemic stroke, rather than establish causality. The inherent limitations of observational research, such as unmeasured confounding factors and potential reverse causality, may influence the interpretation of the results. Second, the GBD estimates rely on modeled data whose accuracy may be compromised in areas with limited primary data, potentially influencing subnational-level analyses. Third, although the high BMI definition (≥23 kg/m²) follows GBD China-specific criteria, employing different cutoffs could produce marginally divergent results. Fourth, the ARIMA-based projection models operate under the assumption of persistent historical trends and linearity, thus failing to incorporate potential modifying factors such as future policy reforms, medical innovations, or socioeconomic crises that may impact BMI or IS epidemiology.

## Conclusion

This study reveals the continuous increase in the burden of high BMI-related ischemic stroke in China and the gender differences therein. This trend may be associated with factors such as China’s economic development, urbanization process, changes in living habits, population aging, and public health policies. The striking contrast with the continuous decline in the burden of IS in high-income countries underscores the urgent need for targeted intervention strategies in China, such as launching public science initiatives on weight loss, establishing additional weight loss clinics in medical institutions, increasing funding support for weight loss-related scientific research, and encouraging the research and development of weight loss medications. Given that the burden may further escalate in the future, proactive policy formulation is likely to help prevent the further prevalence of IS.

## Supporting information

S1 File Revised manuscript with track changes. (DOCX)
